# Increased left ventricular mass index is present in patients with type 2 diabetes without ischemic heart disease

**DOI:** 10.1038/s41598-018-19229-w

**Published:** 2018-01-17

**Authors:** Jelena P. Seferovic, Milorad Tesic, Petar M. Seferovic, Katarina Lalic, Aleksandra Jotic, Tor Biering-Sørensen, Vojislav Giga, Sanja Stankovic, Natasa Milic, Ljiljana Lukic, Tanja Milicic, Marija Macesic, Jelena Stanarcic Gajovic, Nebojsa M. Lalic

**Affiliations:** 1 Clinic of Endocrinology, Diabetes and Metabolic disorders, Clinical Center of Serbia, Dr. Subotica 13, 11000 Belgrade, Serbia; 2 Clinic of Cardiology, Clinical Center of Serbia, Koste Todorovica 8, 11000 Belgrade, Serbia; 3 Department of Cardiology, Herlev and Gentofte Hospital, University of Copenhagen, Copenhagen, Denmark; 4 Center for Medical Biochemistry, Clinical Center of Serbia, Visegradska 26, 11000 Belgrade, Serbia; 5 Institute of Medical Statistics, Faculty of Medicine, Dr. Subotića 15, Belgrade, Serbia; 6 Present Address: University of Belgrade, Faculty of Medicine, Belgrade, Serbia

## Abstract

Left ventricular mass index (LVMI) increase has been described in hypertension (HTN), but less is known about its association with type 2 diabetes (T2DM). As these conditions frequently co-exist, we investigated the association of T2DM, HTN and both with echocardiographic parameters, and hypothesized that patients with both had highest LVMI, followed by patients with only T2DM or HTN. Study population included 101 T2DM patients, 62 patients with HTN and no T2DM, and 76 patients with T2DM and HTN, excluded for ischemic heart disease. Demographic and clinical data, biochemical measurements, stress echocardiography, transthoracic 2D Doppler and tissue Doppler echocardiography were performed. Multivariable logistic regression was used to determine the independent association with T2DM. Linear regression models and Pearson’s correlation were used to assess the correlations between LVMI and other parameters. Patients with only T2DM had significantly greater LVMI (84.9 ± 20.3 g/m^2^) compared to patients with T2DM and HTN (77.9 ± 16 g/m^2^) and only HTN (69.8 ± 12.4 g/m^2^). In multivariate logistic regression analysis, T2DM was associated with LVMI (OR 1.033, 95%CI 1.003–1.065, p = 0.029). A positive correlation of LVMI was found with fasting glucose (p < 0.001) and HbA1c (p = 0.0003). Increased LVMI could be a potential, pre-symptomatic marker of myocardial structural change in T2DM.

## Introduction

Cardiovascular diseases (CV) are the leading cause of morbidity and mortality in patients with type 2 diabetes mellitus (T2DM)^1^. Also, T2DM is considered a major co-morbidity and a strong independent risk factor for the development and progression of heart failure (HF)^2^, with both preserved or reduced ejection fraction (EF)^3^. It has been shown that worse glycemic control has been associated with worsening of cardiac structure and function^4^.

There is evidence suggesting that T2DM could be associated with increased left ventricular (LV) mass^5,6^, concentric geometry/remodeling, and impaired diastolic function^7^. Over time, these structural and functional changes result in impaired systolic function and symptomatic HF, which are associated with worse clinical outcomes^8^. Hypertension (HTN) has previously been associated with elevated LV mass^9^. Also, it is well known that LV mass increases with age, obesity, dyslipidemia, which are often present in T2DM and HTN^10,11^. Therefore, these patients have a higher incidence of increased LV mass, as well as other myocardial impairments, in comparison to patients without multiple co-morbidities^12–14^.

We therefore investigated the association of T2DM, HTN and both co-morbidities with LV mass measured by echocardiography. We hypothesized that patients with both T2DM and HTN had highest LV mass, followed by patients with only T2DM or HTN.

## Methods

The study population included 101 T2DM patients with normal blood pressure (BP), 62 patients with HTN and no T2DM, and 76 patients with T2DM and HTN. Ischemic heart disease was excluded in all patients. None had prior history or symptoms of heart failure (chest pain, dyspnea, arrhythmia and synkopee), cerebrovascular or renal disease, microvascular diabetic complications or insulin therapy. T2DM was diagnosed based on laboratory data (glycated hemoglobin (HbA1c) ≥6.5%), medical history of T2DM or therapy (sulfonylurea and/or metformin). HTN was diagnosed using BP levels higher than 140/90 mmHg or antihypertensive treatment (renin–angiotensin system inhibitors, angiotensin II–receptor antagonist, calcium channel blockers, diuretics and beta-blockers, alone or in combination)^15^. Patients were prospectively recruited from the Clinic of Endocrinology, Diabetes and Metabolic Disorders and Clinic of Cardiology, Clinical Center of Serbia, between October 2007 and May 2013.

Demographic and clinical data, as well as anthropometric, echocardiographic and biochemical measurements were obtained for all patients. The study protocol was in adherence to the contents of the Declaration of Helsinki. Informed consent was obtained for all participants and the Medical Ethical Committee of the Clinical Center of Serbia approved the study protocol.

Blood samples were collected following 12-hour fasting [serum glucose, HbA1c, lipid parameters, creatinine], and were analyzed using standard methods. Insulin was assessed by radioimmunoassay method using a commercially available kit. Kidney function was evaluated using the Modification of Diet in Renal Disease Study equation (MDRD) for estimating glomerular filtration rate with standardized serum creatinine^16^. Albumin/creatinine ratio was done in a first morning urine sample. Insulin sensitivity was estimated by Homeostasis Model Assessment of Insulin Resistance (HOMA-IR)^17^, using the formula HOMA-IR = insulin (mU/l) × glycaemia (mmol/l)/ 22.5. Antihiperglycemic therapy was discontinued 48–72 hours prior to blood sampling. Body mass index (BMI) was calculated using the following formula weight (kg)/height (m)^2^. BP was measured in supine position with a cuff adjusted to arm circumferential after at least 5 minutes of rest. Stress echocardiography test was used to exclude ischemic heart diseases (Del Mar and Agilent Image Point, USA) using the Bruce protocol^18^. Myocardial ischemia was defined as occurrence of new wall motion abnormality (hypokinesia/akinesia). All patients underwent transthoracic 2D Doppler and tissue Doppler echocardiography using Sequoia c256 Acuson (Siemens Mountain View, California, USA). Standard two dimensional, M-Mode, pulsed Doppler measures were done according to the updated Recommendations for cardiac chamber quantification by echocardiography in adults^19^. Atrial volumes and ejection fraction (EF) were assessed using the modified Simpson biplane method^19^. LV mass was calculated using the Devereux formula and normalized by body surface area (LV mass index [LVMI])^20^. Relative wall thickness (RWT) was calculated as 2 times PW divided by the LV diastolic diameter^21^. Early and late diastolic peak filling velocities E and A wave were measured at the mitral leaflet tips. The early (e’) and late (a’) diastolic velocities at septal and lateral corner of mitral annulus were assessed with pulse-wave Tissue Doppler from a standard apical 4-chamber view^19^. Filters were set to exclude high frequency signals, while direction of annulus motion was aligned with the scan line direction. An experienced investigator, blinded for the clinical data, performed and interpreted all echo-Doppler recordings.

### Statistical analysis

Baseline characteristics, laboratory analysis and echocardiographic data were stratified by the presence of T2DM, HTN or both. Descriptive data is presented as the mean ± standard deviation for normally distributed variables and as median [25–75th percentile] for non-normally distributed variables. Categorical variables are expressed as absolute numbers with percentages and were compared by the χ^2^-test, while continuous variables were compared using univariate one-way ANOVA or Kruskal-Wallis analysis of variance, as appropriate. Multivariable logistic regression model including all significantly different baseline characteristics and echocardiographic parameters was used to determine the independent association with T2DM. Linear regression models and Pearson’s correlation were used to assess the correlations between LVMI and other parameters. Two-sided P-values < 0.05 were considered significant. Analyses were performed using SPSS Windows version 21.0.

### Data availability

The datasets used and/or analyzed during the current study available from the corresponding author on reasonable request.

## Results

The study population included 239 patients (51.5% males, mean age 55.4 ± 8.5 years): 101 T2DM and no HTN, 62 HTN and no T2DM, and 76 patients with both T2DM and HTN. All patients were excluded for ischemic heart disease. Patient characteristics and baseline laboratory data are presented in Table 1. Patients who had only T2DM were predominantly male, and 48% had a family history of T2DM. Also, they had higher fasting glucose, insulin, HOMA-IR and used metformin and statin more frequently. Although both were within normal ranges, creatinine was significantly higher, while eGFR was lower in these, compared to other patients. Patients who had both T2DM and HTN were older, with a significantly longer duration of T2DM, higher BMI, HbA1c and triglycerides.

**Table 1:** Baseline characeristics and laboratory data.

Parameter	T2DM, no HTN* n = 101	HTN, no T2DM** n = 62	T2DM and HTN*** n = 76	p	Post hoc multiple comparisons
Age, years	54.5 ± 9.0	54.3 ± 8.0	57.4 ± 7.8	0.04	
Male sex, n (%)	64 (63)	21 (34)	38 (50)	0.001	*vs**
Duration of disease, years					
T2DM	4.0 (2.0–5.0)		5.0 (3.0–10.0)	<0.001	
HTN		5.0 (2.0–8.0)	5.0 (2.0–8.0)	0.23	
Family history, n (%)					
T2DM	48 (48)	16 (26)	33 (43)	0.02	*vs**
CVD	40 (40)	49 (79)	48 (63)	<0.001	*vs**; *vs***
Smoking, n (%)	37 (37)	27 (44)	18 (24)	0.04	**vs***; *vs***
Body mass index, kg/m^2^	26.7 (24.7–29.5)	25.6 (23.7–27.7)	28.9 (26.0–30.9)	<0.001	**vs***
Blood pressure, mmHg					
Systolic	130 (120–130)	150 (145–150)	150 (145–150)	<0.001	*vs**; *vs***
Diastolic	80 (75–80)	95 (95–95)	95 (95–100)	<0.001	*vs**; *vs***
Medications, n (%)					
Metformin	97 (96)		67 (88)	0.05	
Sulfonylurea	52 (52)		47 (62)	0.17	
Statin	24 (24)	11 (18)	7 (9)	0.04	*vs***
ACE inhibitor		42 (68)	60 (79)	0.14	
Angiotensin II receptor blocker		4 (7)	4 (5)	0.77	
Diuretic		20 (32)	11 (15)	0.01	
Calcium antagonist		25 (40)	32 (42)	0.83	
Beta blocker		31 (50)	33 (43)	0.44	
Fasting glucose, mmol/l	7.9 (6.7–9.1)	5.5 (5.0–5.9)	7.8 (6.3–9.0)	<0.001	*vs**; **vs***
Glycated hemoglobin, %	7.2 ± 1.0	5.6 ± 0.6	7.4 ± 1.1	<0.001	*vs**; **vs***
Insulin, mU/L	16.7 (11.5–24.6)	10.4 (7.0–15.9)	11.9 (7.3–16.6)	<0.001	*vs**; *vs***
HOMA-IR	5.3 (4.0–8.4)	2.5 (1.7–4)	3.7 (2.3–5.6)	<0.001	*vs**; *vs***
Total cholesterol, mmol/l	5.7 ± 1.3	6.2 ± 1.1	5.8 ± 1.2	0.03	*vs**
LDL cholesterol, mmol/l	3.6 ± 1.1	4.1 ± 0.9	3.4 ± 1.2	0.002	*vs**; **vs***
HDL cholesterol, mmol/l	1.1 ± 0.3	1.4 ± 0.3	1.2 ± 0.3	<0.001	*vs**; **vs***
Triglycerides, mmol/l	1. 9 (1.4–2.7)	1.4 (1.1–1.8)	1.9 (1.5–2.8)	<0.001	*vs**; **vs***
Creatinine, μmol/l	83.1 ± 15.4	69.7 ± 12.1	70.5 ± 15	<0.001	*vs**; *vs***
eGFR, ml/min/1.73 m^2^	83 ± 18.7	92 ± 14.5	95.5 ± 20.2	<0.001	*vs**; *vs***

ANOVA; Data is presented as means ± SD, median [25–75th percentile], or percentages; T2DM-type 2 diabetes, HTN-hypertension, CVD-cardiovascular diseases; HOMA-IR-homeostatic model assessment of insulin resistance index, HDL-high density lipoprotein, LDL-low density lipoprotein, eGFR-estimated glomerular filtration rate.

Echocardiographic parameters are presented in Table 2. Although systolic function was normal in all patients, EF was significantly lower, while left ventricular end systolic and end diastolic diameter, as well as stroke volume, were significantly higher in patients with only T2DM. LV mass and LVMI were highest in patients with T2DM, followed by patients with both co-morbidities and were lowest in patients with only HTN. Deceleration time was shortest in T2DM patients, followed by the HTN group and patients with both co-morbidities. The differences in echocardiographic parameters among groups remained significant when evaluated by gender (Supplemental Table 1).

**Table 2:** Echocardiographic parameters.

Parameter	T2DM, no HTN* n = 101	HTN, no T2DM** n = 62	T2DM and HTN*** n = 76	p	Post hoc multiple comparisons
EDD, mm	51.0 ± 4.5	47.6 ± 3.4	49.3 ± 3.8	<0.001	*vs**; *vs***
ESD, mm	32.5 ± 4.5	29.7 ± 2.9	30.8 ± 3.1	<0.001	*vs**; *vs***
Stroke volume, ml	78.7 ± 16.5	71.39 ± 14.0	77.1 ± 14.2	0.013	*vs**
Ejection fraction, %	65.3 ± 6.4	67.5 ± 4.7	67.2 ± 4.7	0.020	*vs**
LAVI, ml/m^2^	25.6 ± 6.2	25.8 ± 6.9	24.6 ± 5.8	0.43	
Left ventricular mass, g	167.4 ± 41.6	131.3 ± 28.3	152.3 ± 36.1	<0.001	*vs**; *vs***; **vs***
LVMI, g/m^2^	84.9 ± 20.3	69.8 ± 12.4	77.9 ± 16	<0.001	*vs**; *vs***; **vs***
Cardiac index, l/min/m^2^	3.1 ± 0.7	2.8 ± 0.6	3.2 ± 0.9	0.06	
E, m/s	0.61 ± 0.15	0.63 ± 0.14	0.62 ± 0.15	0.65	
A, m/s	0.68 ± 0.16	0.69 ± 0.13	0.76 ± 0.17	0.002	*vs***; **vs***
E/A	0.85 (0.71–1.15)	0.88 (0.7–1.14)	0.76 (0.69–0.92)	0.039	*vs***
DT, msec	216.3 ± 47.9	228.4 ± 39.1	233.8 ± 47.0	0.033	*vs***
E/E’ mean	5.56 ± 1.7	5.65 ± 1.39	5.56 ± 2.0	0.94	
Relative wall thickness	0.32 ± 0.07	0.30 ± 0.04	0.32 ± 0.05	0.14	

ANOVA; Data is presented as means ± SD, median [25–75th percentile], or percentages; T2DM-type 2 diabetes, HTN-hypertension; EDD-left ventricular end diastolic diameter, ESD-left ventricular end systolic diameter, LAVI-left atrial volume index, LVMI-left ventricular mass index, E-early mitral valve flow velocity, A-late mitral valve flow velocity, E/A-ratio of early to late mitral valve flow velocity, DT-deceleration time, E/E′-ratio of early mitral valve flow velocity to early Tissue Doppler lengthening velocity.

**Figure 1:**
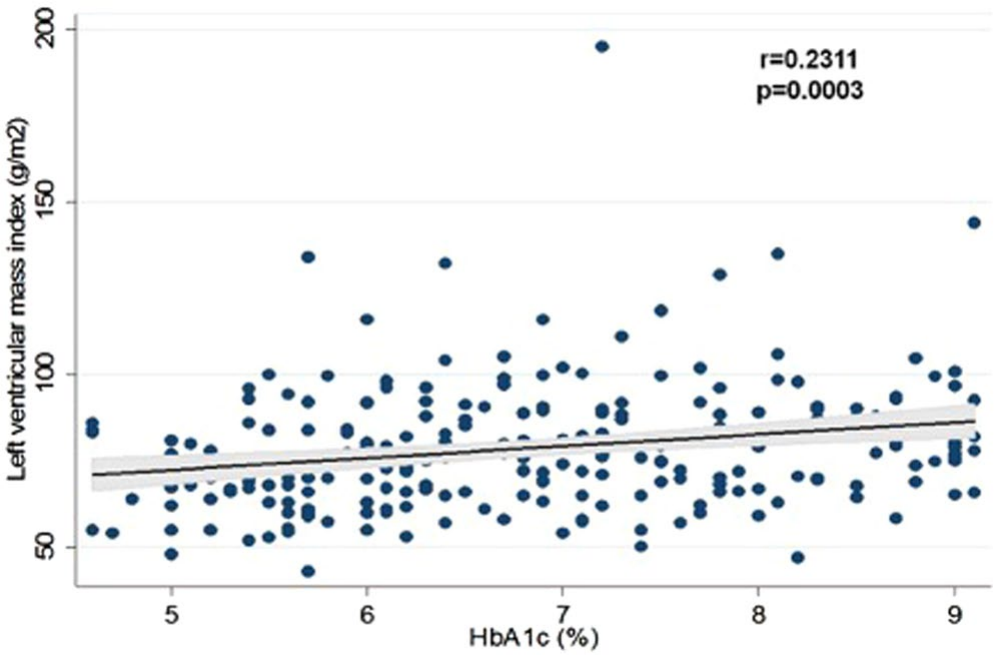
Correlation of left ventricular mass index and glucose.

**Figure 2:**
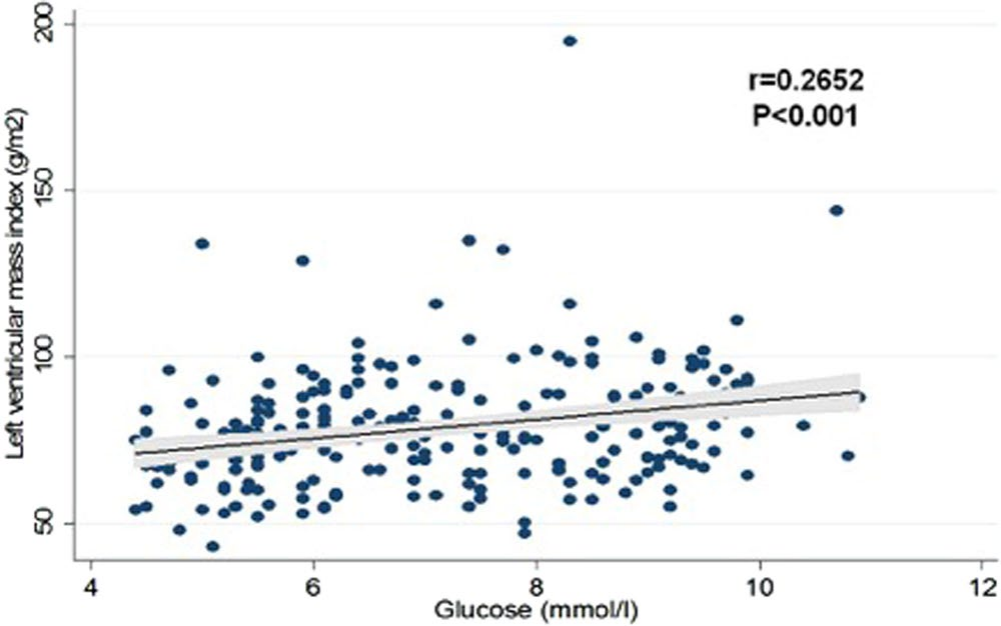
Correlation of left ventricular mass index and HbA1c.

In multivariable logistic regression analysis, T2DM was associated with BMI (OR = 1.200, 95%CI 1.070–1.347, p = 0.002), diastolic BP (OR = 0.847, 95%CI 0.768–0.934, p = 0.001), and LVMI (OR 1.033 95%CI 1.003–1.065, p = 0.029; Table 3). In multivariable linear regression analysis LVMI was associated with T2DM (standarized β = 0.143, p = 0.037), male sex (standarized β =−0.254, p < 0.001), mean E/E’ ratio (standarized β = 0.268, p < 0.001), hypertension (standarized β = −0.179, p = 0.009) and E/A ratio (standarized β =−0.143, p = 0.016; Table 4). Also, a positive correlation of LVMI was found with fasting glucose, HbA1c, and creatinine. In addition, LVMI correlated with male sex, HDL cholesterol, systolic and diastolic blood pressures, and eGFR (Table 5, Figs 1 and 2).

**Table 3:** Multivariable analysis of baseline characteristics and echocardiographic parameters associated with type 2 diabetes.

**Parameter**	**OR**	**95% Confidence interval**	**p**
Age, years	1.052	0.999–1.109	0.05
Female sex	0.595	0.263–1.345	0.21
Body mass index, kg/m^2^	1.200	1.070–1.347	0.002
Systolic blood pressure, mmHg	0.998	0.932–1.069	0.96
Diastolic blood pressure, mmHg	0.847	0.768–0.934	0.001
Ejection fraction, %	0.956	0.885–1.034	0.26
LVMI, g/m^2^	1.033	1.003–1.065	0.029
E/A	0.828	0.150–4.554	0.83
DT, msec	0.994	0.985–1.003	0.19
E/E’ mean	0.978	0.768–1.244	0.86

LVMI-left ventricular mass index, E/A-ratio of early to late mitral valve flow velocity, DT-deceleration time, E/E′-ratio of early mitral valve flow velocity to early Tissue Doppler lengthening velocity.

**Table 4:** Multivariable analysis of baseline characteristics and echocardiographic parameters associated with left ventricular mass index.

Parameter	β coefficient	SE	β standarized	p
Type 2 diabetes	5.898	2.806	0.143	0.037
Female sex	−9.200	2.184	−0.254	<0.001
E/E’ mean	2.814	0.617	0.268	<0.001
Hypertension	−6.552	2.491	−0.179	0.009
E/A	−9.604	3.960	−0.143	0.016

LVMI-left ventricular mass index, E/A-ratio of early to late mitral valve flow velocity, E/E′-ratio of early mitral valve flow velocity to early Tissue Doppler lengthening velocity.

**Table 5:** Correlations of left ventricular mass index and baseline characteristics.

	Parameter	r	p
Left ventricular mass index, g/m^2^	Female sex	−0.2721	<0.001
	Glucose, mmol/l	0.2652	<0.001
	Glycated hemoglobin, %	0.2311	0.0003
	HDL cholesterol, mmol/l	−0.2089	0.001
	Creatinine, μmmol/l	0.3186	<0.001
	eGFR, ml/min/1.73 m^2^	−0.1410	0.030
	Systolic blood pressure, mmHg	−0.2281	0.0004
	Diastolic blood pressure, mmHg	−0.2408	0.0002

r-Pearson’s correlation coefficient; HDL-high density lipoprotein; eGFR-estimated glomerular filtration rate.

## Discussion

In our study, patients with T2DM and no HTN, free of ischemic heart disease, had significantly larger LVMI in comparison to patients with both T2DM and HTN, and those with only HTN. In multivariable analysis, T2DM was significantly associated with LVMI. In addition, increasing LVMI was positively associated with fasting glucose and HbA1c.

In the real clinical setting, patients with T2DM usually have co-existing HTN, both known to contribute to the increase of LVMI. Therefore, we expected to find the highest LVMI in that group, which was not the case. HTN per se is a well-known cause of LVM increase^8,22–24^. However, in our study, investigated patients with only HTN had smallest LVMI. Our results are in contrast to the Strong Heart Study, which showed that the combination of T2DM and HTN lead to the highest LVMI, followed by patients with only HTN and T2DM, respectively^25^. Although a long asymptomatic period may precede T2DM symptoms and diagnosis, and reported duration of the disease may be underestimated, patients included in our study were considered to be at the beginning of cardio-metabolic continuum and early phase of myocardial impairment. Even so, largest LVMI in this group indicates the possible negative effect of hyperglycemia on LV mass increase, even before overt T2DM. Furthermore, there is data supportive of the link between pre-diabetic states (insulin resistance, impaired fasting glucose and impaired glucose tolerance) with increase in LV mass^25–27^. Also, worse glycemic control reflected in higher HbA1c has been associated with LV mass increase^28,29^, which is in line with our results. In a large community-based cohort, participants with T2DM had a steeper increase in LV mass over time compared to those without T2DM^30^.

It has been suggested that T2DM induces LV mass enlargement through “metabolic”, and not hemodynamic pathways^31,32^. Hyperglycaemia and hyperinsulinemia cause interstitial deposition of advanced-glycated end products, increased serum aldosterone levels causing myocyte growth and changes in the extracellular matrix, and activation of cytokines and angiotensin II, all leading to myocardial fibrosis and subsequent increase in LV mass^33,34^. Hence, LVMI increase seems to be present in T2DM despite no increase in afterload, suggesting the existence of an isolated effect of T2DM on the structural remodeling of the heart. In the Strong Heart Study, early cardiac impairment in T2DM was characterized by increased LV mass and subclinical LV dysfunction^35^. In our T2DM cohort, the only signal of cardiac impairment was increased LVMI, while diastolic and systolic function were normal. We therefore speculate that increase in LV mass could be an early marker of cardiac dysfunction in T2DM patients, developing even prior to the asymptomatic diastolic dysfunction. Timely identifying increase in LV mass is very important, as it has been shown to be a strong predictor of sudden cardiac death^36,37^, CV disease^38^, and all-cause mortality in both middle-aged^39^, and elderly individuals^40^. A similar notion has been proposed by Levy *et al*., who suggested that LV mass provides additional prognostic information to the traditional CV risk factors in the general population^41^. Therefore, the results of the current study uphold the European Society of Cardiology and European Association for the Study of Diabetes Guidelines on diabetes, pre-diabetes, and cardiovascular diseases^42^ which suggest that echocardiography should be considered as a regular screening tool in all T2DM patients, even if they are asymptomatic and without overt cardiovascular disease^43,44^. This interesting finding points out the importance of identifying patients at high risk for CV events and also sets the stage for the future investigation which should determine the role of LVMI as a potential independent CV risk factor.

Several limitations of current study need to be mentioned. This analysis was cross-sectional, and therefore no conclusions on causality could be drawn. Coronary angiography was not performed, due to the invasiveness and cost, hence all patients underwent stress echocardiography, which is considered to have the sensitivity of 85% and specificity of 77% for the detection of coronary artery disease^45^. Glucose clamp, the gold standard in the assessment of insulin sensitivity was not used in this study. As HOMA-IR was previously shown to strongly correlate with clamp-measured insulin resistance, we used this reliable and applicable diagnostic tool.

## Conclusions

LVMI was largest in patients with only T2DM, which suggests that it could be a potential, pre-symptomatic marker of myocardial structural change in T2DM. Also, LVMI was associated with higher fasting glucose and HbA1c, indicating the possible role of hyperglycemia in LV mass increase.

## Electronic supplementary material

Supplemental Table 1
