# Supplementary material for: Increased left ventricular mass index is present in patients with type 2 diabetes without ischemic heart disease
Source: Sci Rep. 2018 Jan 17;8:926. doi: 10.1038/s41598-018-19229-w (PMC5772487; doi:10.1038/s41598-018-19229-w)
Supplement: Supplementary file 1 — Supplemental Table 1 [file 41598_2018_19229_MOESM1_ESM.doc]

**Supplemental material**

**Increased left ventricular mass index is present in patients with type 2 diabetes without ischemic heart disease**

Jelena P. Seferovic^1^*, Milorad Tesic^2^*, Petar M. Seferovic^2^*, Katarina Lalic^1^*, Aleksandra Jotic^1^*, Tor Biering-Sørensen^3^, Vojislav Giga^2^*, Sanja Stankovic^4^, Natasa Milic^5^, Ljiljana Lukic^1^*, Tanja Milicic^1^*, Marija Macesic^1^*, Jelena Stanarcic Gajovic^1^, Nebojsa M. Lalic^1^*

**Supplemental Table 1. Echocardiographic parameters, by gender**

| **Parameter** | **T2DM, no HTN** | **HTN, no T2DM** | **T2DM and HTN** | **p** |
| --- | --- | --- | --- | --- |
| EDD, mm |  |  |  |  |
| male | 52.7±3.7 | 50.6±2.3 | 50.7±4.0 | 0.01 |
| female | 48.2±4.3 | 46.1±2.9 | 47.8±3.1 | 0.022 |
| ESD, mm |  |  |  |  |
| male | 33.8±3.9 | 31.2±2.7 | 32.0±3.1 | 0.003 |
| female | 30.1±4.6 | 28.9±2.7 | 29.6±2.5 | 0.33 |
| Stroke volume, ml |  |  |  |  |
| male | 84.0±16.1 | 82.5±10.8 | 81.6± 15.3 | 0.75 |
| female | 70.7±14.1 | 65.7±119 | 72.6± 11.7 | 0.044 |
| Ejection fraction, % |  |  |  |  |
| male | 64.1±6.2 | 67.9±5.4 | 66.3±5.2 | 0.019 |
| female | 67.5±6.3 | 67.4±4.3 | 68.1±4.0 | 0.79 |
| LAVI, ml/m^2^ |  |  |  |  |
| male | 26.2±5.9 | 26.8±6.1 | 24.2±5.0 | 0.14 |
| female | 24.6±6.6 | 25.3±7.3 | 25.0±6.6 | 0.90 |
| Left ventricular mass, g |  |  |  |  |
| male | 178.6±37.9 | 152.4±25.8 | 169.4±35.0 | 0.013 |
| female | 147.9±40.9 | 120.5±23.2 | 135.1±28.6 | <0.001 |
| LVMI, g/m^2^ |  |  |  |  |
| male | 87.5±17.9 | 74.3±14.3 | 82.0±15.3 | 0.006 |
| female | 80.3±23.5 | 67.6±10.7 | 73.9±16.0 | 0.007 |
| Cardiac index, l/min/m^2^ |  |  |  |  |
| male | 3.1±0.8 | 3.0±0.6 | 3.1±0.9 | 0.84 |
| female | 3.1±0.8 | 2.8±0.6 | 3.2±0.8 | 0.06 |
